# Infectious Diseases in the Context of the War in Ukraine: Refugee Health Implications in Romania

**DOI:** 10.3390/healthcare13212732

**Published:** 2025-10-28

**Authors:** Olga Adriana Caliman-Sturdza, Roxana Gheorghita, Monica Terteliu Baitan, Roxana Filip

**Affiliations:** 1 Faculty of Medicine and Biological Sciences, Stefan cel Mare University of Suceava, 720229 Suceava, Romania; olga.caliman-sturdza@usm.ro (O.A.C.-S.); monica.terteliu@usm.ro (M.T.B.); roxana.filip@usm.ro (R.F.); 2 Suceava Emergency Clinical County Hospital, 720237 Suceava, Romania

**Keywords:** infectious diseases, migrants, Ukraine, healthcare access, accessibility

## Abstract

Background: Refugees often face major health challenges owing to displacement, poor living conditions, limited access to healthcare, and the psychological toll of forced migration. Access to healthcare has been a major concern because of disrupted medical services, pre-existing health conditions, and integration challenges in host countries. This study aimed to evaluate the effect of infectious diseases on refugees in the context of the war in Ukraine by analyzing data from patients who accessed health services from a county hospital. Methods: We analyzed the data of Ukrainian refugees who presented for an infectious disease between February 2022 and March 2025 in the largest hospital unit in Romania located immediately near the border with Ukraine. Results: A total of 2052 refugee patients of Ukrainian nationality presented to the Emergency Reception Unit of “St. Ioan cel Nou Suceava” for consultations; 672 patients required an evaluation by an infectious disease specialist and 48 were hospitalized in the Department of Infectious Diseases. The most common disease encountered in children was influenza, whereas the most common disease in adults was SARS-CoV-2 infection. The most frequently encountered comorbidities in pediatric patients were anemia (26.9%) and dehydration syndrome (46.2%). In adults, comorbidities included chronic obstructive pulmonary disease (18.2%), hypertension (13.6%), chronic coronary disease (4.5%), diabetes (9.1%), and chronic hepatitis (4.5%). Patients were treated with antivirals, rehydration solutions or only symptomatic treatment. Conclusions: Romania is implementing public health measures to address these challenges, focusing on vaccination and disease screening, and ensuring access to essential healthcare services. These services include access to primary care physicians, specialist consultations, hospitalization, and essential medications.

## 1. Introduction

**Figure 1:**
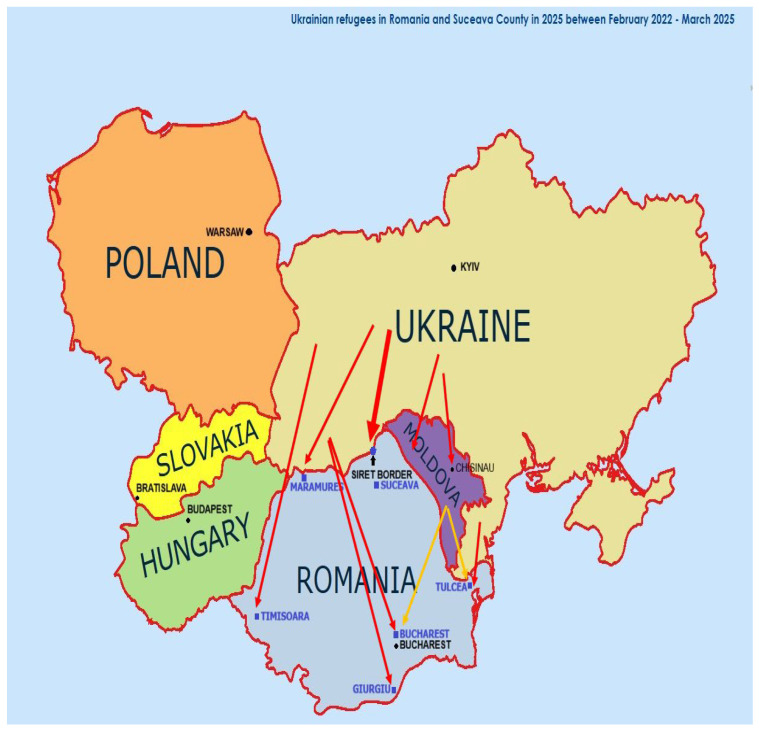
Ukrainian refugees recorded in Romania and Suceava County (February 2022–March 2025); (Red Arrows show the main settlement areas of Ukrainian refugees).

Romania has substantially supported Ukrainian refugees since Russia invaded Ukraine in February 2022. As of 31 March 2025, approximately 182,948 Ukrainian refugees were recorded in Romania [1] and since 24 February 2022, over 5.8 million border crossings from Ukraine to Romania have been recorded [2,3] (Figure 1).

The Romanian government, in collaboration with civil society and international organizations, has implemented various measures to assist refugees, granting them access to essential services and support [4,5]. The government has also established six refugee centers in Suceava, Bucharest, Giurgiu, Timișoara, Maramureș, and Tulcea, offering accommodation and meals [6]. Although immediate relief is essential, long-term integration remains a priority. Studies exploring the perceptions and social challenges faced by Ukrainian refugees in Romania highlight the importance of awareness, solidarity, and comprehensive support systems [7]. These efforts aim to facilitate smoother integration into various aspects of daily life, ranging from education to employment. To address the educational needs of Ukrainian children, Romania implemented policies to facilitate their enrollment in local schools. In April 2023, the government enacted an ordinance that made school enrollment a prerequisite for accessing humanitarian assistance, leading to a marked increase in Ukrainian children’s participation in the Romanian education system [8]. The Romanian government, in collaboration with civil society and international organizations, implemented various measures to assist these individuals [9]. Romania’s efforts reflect a comprehensive approach to support Ukrainian refugees by encompassing governmental policies, educational integration, and community-driven initiatives [10]. Ukrainian refugees in Romania are entitled to medical services equivalent to those provided to insured Romanian citizens, including access to primary care physicians, specialist consultations, hospitalizations, and essential medications [11]. Notably, refugees are exempt from social health insurance contributions and co-payments for outpatient treatments [12]. Furthermore, specialist outpatient services are available without referral from a primary care doctor. Medical services accessible to Ukrainian refugees in Romania are funded by the state.

From the first days of the refugee influx psychosocial support has been a priority to help children deal with trauma and displacement [13]. In 2022 child-friendly spaces were established in transit areas and in refugee centers (e.g., in Bucharest) to offer a safe and playful space for children as well as psychological first aid for children exhibiting signs of distress. Mobile teams of social workers and psychologists facilitated child play activities, paid attention to the emotional needs of the children and provided counseling for the children and the mothers [13]. For example, the Non Guvernamental Organization (NGO) Terre des Hommes (Tdh) opened an Innovation and Resilience Center (RIF) in Bucharest (2022–2023), where the Ukrainian children have emotional support through creative workshops and mentorship and access to on-site psychologists for emotional counseling [14]. In March 2022, UNICEF and UNHCR, in partnership with local authorities, started opening “Blue Dot” hubs for children and families on the border crossings and major cities in Romania. Blue Dots are safe places providing free and integrated services for refugee children and families [15]. They offer trauma-informed care such as psychological therapy and counseling, child-friendly play areas and mother and baby rooms for infant care. These hubs also provide information and referrals (for health problems, cases of violence, etc), basic first aid in health and hygiene, legal advice as well as distribution of basic items such as blankets, hygiene kits, toys, and baby food. As of 2023, Blue Dot centers were opened in Romania at all main border points of Ukraine (e.g., Siret, Sighetu Marmației, Isaccea) as well as in Brașov and Bucharest, thus excluding any families from the country. Through these hubs and other initiatives, UNICEF and partners were able to provide protection as well as mental health services to thousands—more than 230,000 children and women accessed such support in Romania [16]. A variety of actors have deployed mental health and psychosocial support (MHPSS) programs. For example, WHO Romania trained cultural mediators who were escorting refugees to healthcare visits and offering psychosocial support by April 2024. In a month’s time (July 2025), WHO’s mediators provided 79 individual and group counseling sessions that featured art therapy activities, where 242 Ukrainian refugees were reached with the focus on emotional coping and resilience [17]. The International Organization for Migration (IOM) Romania also implemented MHPSS programs in collaboration with local organizations and a network of Ukrainian speaking psychologists: group and individual counseling, art and theater therapy workshops and non-formal educational activities for both children and parents [18]. Many initiatives combine aspects of psychosocial care and integration. NGOs such as Save the Children Romania set up “Teenager’s and Children’s Clubs” in which young people from both Ukraine and Romania meet on a regular basis and support each other as well as social cohesion [19]. Tdh also started a Child Advisory Board for refugee teenagers to express their needs and participate in community life, which empowers them in the recovery process [20]. The Romanian Red Cross has also played a part: Over the course of July 2025, the Red Cross has provided support and services for 505 refugee children and adults through recreational and skill-building workshops, sports and teamwork activities—structured activities designed to strengthen mental health and rebuild community bonds among displaced families [21]. In Romania, there are also programs for Ukrainian refugee mothers, including pregnant women, with services ranging from prenatal care to postpartum support (Table 1).

Before the war, Ukraine had a hospital-based care system that provided relatively high bed density by European standards (~6.3 beds/1000 persons in 2020), but which was characterised by high prevalence of chronic non-communicable diseases (NCD), the underfunding and out-of-pocket payments [26]. Since 2022 the system has been systematically attacked and disrupted: WHO verified 1940 attacks on healthcare by August 2024 and >2250 by February 2025; hundreds of facilities are damaged/looted and health workers have left affected areas [27]. The system of Romania is EU-integrated, with universal coverage, but has lower indicators of spending and access than the EU: (2021 spending per capita: EUR 1663; unmet care needs: ~4.9% vs. 2.2% EU, number of physicians per 1000 population: ~3.5 vs. 3.1 EU, number of hospital beds per 1000 population: ~6–7 vs. 17.9 EU) [28]. Employee shortages are a recurrent factor [29]. There were 4.34 million people from Ukraine under temporary protection in the EU up to 31 July 2025. Czechia, Poland and Estonia have the highest per capita number of hosts; Germany and Poland have the largest absolute numbers (~1.2 m and ~1.0 m, respectively) [30]. In an ongoing survey conducted in 2024 in ten countries hosting refugees (including EU countries and Moldova), an estimated 83% of Ukrainian refugees who needed health services in the previous month said they were able to access them. However, this percentage was more in 2023 (~88%) and has slightly reduced (Table 2) [31].

Suceava County in northern Romania has become a critical hub for Ukrainian refugees seeking safety and support since the onset of the conflict in Ukraine in February 2022. The region’s proximity to the Siret border crossing positioned it as a primary entry point for refugees. Numerous Ukrainian families crossed Romania through the Siret border during the early stages of the crisis. Upon arrival, they received immediate assistance from local communities and international organizations, ensuring access to shelter, food, and emotional support [32]. Recognizing the unique needs of children and families, a dedicated space was established at the Siret Border Crossing Point in August 2024. This initiative, a partnership between the Territorial Inspectorate of the Border Police, United Nations Children’s Fund (UNICEF), and United Nations High Commissioner for Refugees, provided a safe and welcoming environment for those entering Romania. This space ensures that mothers and children receive immediate assistance upon arrival [33].

However, the influx of Ukrainian refugees into Romania has raised significant public health concerns, particularly those concerning infectious diseases [34]. Before the conflict, Ukraine faced challenges with diseases such as tuberculosis (TB), measles, polio, hepatitis C, and human immunodeficiency virus (HIV). Notably, as of February 2022, only 36% of Ukrainians were vaccinated against COVID-19, and vaccination rates for other diseases like measles and polio were also low [35]. A study assessing TB among Ukrainian refugees in European countries found that in 2022, 34 countries reported 887 TB cases among individuals born in Ukraine, with 26% of these cases identified as multidrug- or rifampicin-resistant TB. This study emphasizes the importance of adequate TB health services for refugees to ensure proper diagnosis and treatment [36]. Romania has made efforts to provide essential healthcare services to Ukrainian refugees. The World Health Organization (WHO) has established health clinics and supported general practitioner clinics in offering primary healthcare services, including sexual and reproductive health services and mental health support [37]. Cultural mediators have also been employed to facilitate communication between healthcare providers and refugees. However, despite these efforts, several challenges remain. Language barriers, unfamiliarity with the healthcare system, and lack of information have hindered some refugees from accessing medical care [38]. To address these issues, the WHO has disseminated information about healthcare access, vaccinations, and medication availability to refugees [39,40,41]. The influx of refugees poses a challenge to local healthcare systems. “Sf. Ioan Cel Nou” Clinical Emergency Hospital in Suceava is at the forefront of offering emergency care to refugee patients. Medical professionals have addressed various health issues, particularly among children, who represent a vulnerable segment of the refugee population [42]. Hospital experiences highlight the need for adaptable healthcare responses in crises.

Therefore, this study aimed to analyze data regarding the infectious pathology faced by Ukrainian refugees in the Romanian region located at the border with Ukraine.

## 2. Materials and Methods

This study was a retrospective analysis focusing on the incidence of infectious pathologies in refugees in the context of the war in Ukraine. As inclusion criteria in the study, we selected refugee patients of Ukrainian nationality, adults and children (aged 0–18 years), who addressed the Sf. Ioan Cel Nou, Suceava County Emergency Clinical Hospital during the period from the beginning of the war in Ukraine until the end of March 2025 and who had a legal consent. The study included patients who presented to the Emergency Department for an infectious pathology but focused on patients hospitalized in the Infectious Diseases Department. Refugees who did not come from Ukraine and those who presented to the Emergency Unit for non-infectious pathologies were excluded from the study. Saint Ioan cel Nou is a large hospital with broad scope, both emergency and other specialties, and is arguably one of the major medical centers in the Suceava County region, which is adjacent to border areas. Very few hospitals likely match its scale (1200 inpatient beds, 71 day-care beds, 42 companion beds, 31 wards, full range of specialties) in direct border counties. Many border-area hospitals are smaller, with more limited capability. The patients were diagnosed based on clinical, epidemiological, and laboratory data. All hospitalized patients underwent rapid influenza and SARS-CoV-2 antigen (Ag) tests. In some COVID-19 cases, real-time polymerase chain reaction was performed for SARS-CoV-2. Imaging was performed using chest radiography in cases of respiratory diseases such as COVID-19 or influenza, as well as in patients with measles. Inflammatory syndrome was investigated by performing a blood count and determining C-reactive protein levels. Liver and kidney function, blood sugar levels, ionograms, and lactic dehydrogenase levels were also evaluated. In the case of patients presenting with diarrheal syndrome, fecal culture, Campylobacter Ag from fecal matter, toxins for *Clostridioides difficile*, and glutamate dehydrogenase Ag were collected, and a rapid test for rotavirus and adenovirus was performed in children. Blood cultures, sputum cultures, or other secretions were performed for bacteriological diagnosis in some cases. A Ukrainian infectious disease physician or a translator communicated with the patients and their families, thus ensuring that the information provided about the state of health and recommended treatments was fully understood. The data were collected from the patients’ files and the hospital’s IT program, Info-World, by selecting demographic data, established diagnoses, and data on investigations and treatment. The study was approved by the Ethics Committee of “St. Ioan Cel Nou,” Suceava, Romania (approval no. 11/28.02.2025). All adult patients signed an informed consent form upon admission or presentation to the hospital in which they agreed to the processing of data contained in medical documents or in the computer system. For minors, consent was obtained from their parents or legal guardians. Data were analyzed using Microsoft Excel for Microsoft 365 (Microsoft Corp., Redmond, WA, USA).

## 3. Results

Between February 2022 and March 2025, a total of 2052 Ukrainian refugees presented to the Emergency Reception Unit of “St. Ioan cel Nou” Suceava for consultations regarding various medical conditions. Of these, 770 (37.6%) were children and 1282 (62.4%) were adults. Among adults, 51.3% were women and 48.7% were men. Refugee entry into Romania occurred primarily through the Siret border crossing in Suceava County, the most important entry point during the study period. Additional arrivals were registered at refugee centers in Bucharest, Giurgiu, Timișoara, Maramureș, and Tulcea. The geographic distribution of these centers and the main refugee flows into Romania are shown in Figure 1. Most refugees entered Romania directly from Ukraine, although some passed through Moldova. As indicated by the arrows in Figure 1, the thickest flows corresponded to the Siret crossing, with medium-volume flows at other direct entry points and smaller flows through Moldova. A total of 672 patients (32.7%) required evaluation by an infectious disease specialist. The most common diagnoses were COVID-19 (31.0%), upper respiratory tract infections (28.6%), and influenza (19.7%). Less frequent causes included acute enterocolitis, measles, and rare conditions such as HIV complications, mononucleosis, tick bites, and rabies prophylaxis after dog bites (Table 3).

Of the 672 patients evaluated by infectious disease specialists, 48 required hospitalizations in the Department of Infectious Diseases. Children accounted for 27 cases (56.2%), while adults accounted for 21 cases (43.8%). Females were the majority (58.3%) of hospitalized patients. Most hospitalized children were in the 0–14-year age group (Table 4).

The most frequent diagnoses among hospitalized patients were COVID-19 (29.2%) and influenza (29.2%). In children, influenza was predominant (33.3%), whereas adults were more often admitted for COVID-19 (42.9%). Measles (14.8%) and rotavirus enterocolitis (14.8%) were confined to children, while *Cl. difficile* enterocolitis (19.0%), HIV infection (14.3%), and hepatitis C (9.5%) were observed only in adults (Table 5). A child was hospitalized for chickenpox complicated by interstitial pneumonia. An elderly patient was diagnosed with pulmonary sepsis based on clinical (hypotension, tachycardia, oliguria, confusional syndrome), radiological (right lobar pneumonia) and biological data (intense inflammatory syndrome, nitrogen retention, elevated serum lactate, positive blood cultures with *Klebsiella pneumoniae*) and another adult diagnosed with COVID-19 also presented with cellulitis on the right lower limb.

The most frequently encountered comorbidities in pediatric patients were anemia (26.9%) and dehydration syndrome (46.2%). In adults, comorbidities included chronic obstructive pulmonary disease (18.2%), hypertension (13.6%), chronic coronary disease (4.5%), diabetes (9.1%), and chronic hepatitis (4.5%). Imaging suggested that 37.57% of patients with COVID-19 had interstitial or lobular pneumonia, and 75% of patients with measles had interstitial pneumonia. Sputum culture was also performed in patients with radiological images of pneumonia; however, the cultures were sterile in all tested patients. Blood cultures were positive in only one patient diagnosed with pulmonary sepsis, in this case identifying multidrug-resistant *Klebsiella pneumoniae*. The patient was treated according to the antibiogram with meropenem, with favorable clinical and biological evolution.

Adult patients with COVID-19 were treated with antivirals: 13.3% received remdesivir, 40% received favipiravir, and 6.7% received nirmatrelvir/ritonavir. Children diagnosed with SARS-CoV-2 infection received only symptomatic treatment with antithermics, expectorants, and immunomodulators with isoprinosine. Patients with measles were treated with antithermics, vitamin C, and antitussives, and antibiotics were administered in complicated cases of pneumonia. In pediatric cases of enterocolitis caused by rotaviruses, rehydration solutions, antiemetics, antithermic agents, and probiotics were administered. In adult cases of enterocolitis caused by *Cl. difficile*, oral vancomycin was administered for 10 days.

In the two adult patients with chronic viral hepatitis C, antiviral treatment with sofosbuvir/velpatasvir or glecaprevir/pibrentasvir was initiated, and treatment was provided free of charge through the National Program for the Treatment of Viral Hepatitis. In HIV-seropositive patients, clinical, biological, and immunological evaluations were performed to determine the number of CD4+ lymphocytes and plasma viremia levels. Continuity of antiretroviral treatment, initiated in Ukraine through the National Program for Surveillance and Control of HIV/AIDS Infection, was maintained. All patients were discharged in improved condition or fully recovered, with no recorded fatalities. There were only one cases of sepsis requiring admission to the Intensive Care Unit, and hospitalized patients did not develop complications or severe forms of the disease that led to death. Patients with chronic viral hepatitis C or HIV infection continued to receive follow-up care at the Infectious Disease Outpatient Clinic. Regarding hospitalized patients, the average length of hospitalization for each disease ranged between 2.5 and 13 days (Table 6).

## 4. Discussion

The ongoing conflict in Ukraine has resulted in substantial population displacement, heightening concerns regarding the spread of infectious diseases among refugees [43]. The combination of disrupted healthcare services, overcrowded living conditions, and interrupted vaccination programs has increased the vulnerability of this population to various infections [44]. At the outset of the refugee crisis, Ukraine had a high TB burden for European countries. WHO’s estimated incidence of TB in Ukraine in 2021 was around 71/100,000 population with 19,793 cases of TB notified (45.5/100,000) [45]. Particularly worrying was the high rate of drug-resistant TB, multi-drug-resistant TB (MDR-TB) or rifampicin-resistant TB accounted for 31% of new cases of TB and 45% of previously treated cases and approximately 20% of TB patients in Ukraine were co-infected with HIV [46]. These figures made Ukraine one of the countries with a high burden of TB in the European region of the WHO. TB services in Ukraine have been stressed even before 2022, and the war further disrupted TB diagnostic and treatment programs, especially in conflict zones [45,47]. Nonetheless, it seems essential to point out the demographic pattern of TB in Ukraine: the majority of TB cases occurred in adult men (dominant age group 35–44) [48,49]. This epidemiological profile is relevant as 90% of the refugees fleeing Ukraine have been women and children, as most fighting age males were not permitted to leave the country. This disconnect between the profile of the average Ukrainian TB patient and the refugee population is one of the reasons that we are seeing low TB case numbers among refugees. Another key factor is that of vaccination coverage, especially that of the Bacillus Calmette-Guerin (BCG) vaccine which provides partial protection against TB. Ukraine has practiced routine BCG immunization in infants for their national program. In the years just before the war, coverages of BCGs in Ukraine were relatively high (around 85–95% of infants being vaccinated) [50]. Although BCG is not totally effective in preventing pulmonary TB infection, it is effective in preventing serious forms of TB (including miliary TB and TB meningitis in children) and may have some heterologous immune benefits. High BCG coverage means that the majority of young Ukrainian refugees (and many adults that were vaccinated in childhood) had baseline immunity that decreases their risk of developing life-threatening TB. Indeed, the pediatric rate of TB in Ukraine was much lower than the adult rate and the vigorous immune response elicited by the BCG in early life probably contributed to the low rate of active TB in the large number of Ukrainian children who arrived in Romania [46,51]. In Poland, the influx of Ukrainian refugees has contributed to a noticeable increase in the number of TB cases. In 2022, Poland reported 4205 TB cases, including 269 among foreign nationals, of which 175 were Ukrainian citizens [52], representing a doubling of TB cases among foreigners compared with 2021. Similarly, Germany recorded an increase in TB cases among Ukrainian refugees, reporting 2847 TB cases among foreigners in 2022, with 262 involving Ukrainian nationals, an increase of 236 cases from the previous year [53]. The Czech Republic also reported an increase in TB cases among foreigners between 2018 and 2022. In 2022, 156 cases were reported among foreigners, with 87 cases among Ukrainians, more than double that of the previous year [54]. Given that Romania historically has the highest tuberculosis (TB) burden in the EU (48, 7 cases per 100,000 in 2022) there were initial public health concerns about possible importation of TB from Ukraine [55]. Surprisingly, however, relatively few TB cases have been reported in the Ukrainian refugees in Romania 2022–2025 [56].

Although specific TB incidence data among Ukrainian refugees in Romania are limited, the country has implemented screening and treatment programs to manage potential cases. At Suceava County Clinical Hospital, no new cases of TB have been diagnosed among refugee patients since the beginning of the war in Ukraine. According to a survey among 34 European countries, Romania had only 4 TB cases among the people from Ukraine in 2022 [36]. This was out of an estimated 152,621 Ukrainian people living in Romania at the end of 2022 (including both pre-war residents and refugees). In fact, in this population the TB incidence was only 2.6 per 100,000 in 2022, which is much lower than the expected rate (≈44/100 k) as well as than the general population TB rate in Romania [57]. In addition, none of those few cases were reported to be MDR-TB. Early in 2023, Romania only reported 0 new refugees with TB, which indicates the lasting trend of low impact [36]. Unofficial reports through 2024–2025 have not shown any rise in TB among the refugees and numbers are still in the single digits. This is according to Romanian TB experts, that no significant clusters or outbreaks related to the Ukrainian refugees have been registered and the influenza of refugees did not have a measurable impact on overall TB dynamics in the country. The number of TB cases in Romania increased slightly in 2022 (on the back of the post-COVID rebound in detection, rather than the refugees), but this was fairly minor. Several medical and public health reasons exist for why these low numbers of cases are occurring. First, as mentioned, the refugees in Romania were more likely to be from lower risk groups (women, children) and therefore have fewer TB cases. Second, the refugees had high vaccination coverage (BCG) and, in general, a good baseline health status, which decreased the immediate susceptibility. Third, refugees who were already in Ukraine on TB treatment were actively managed: WHO/Europe facilitated connections between host countries and Ukraine’s National TB Program to continue TB treatment for any displaced TB patients [36,58]. This means a Ukrainian refugee already on TB drug treatment can easily continue into treatment in Romania, thus being registered and treated immediately (and unlikely to spread disease). Fourth, many refugees considered Romania a transit country, they stayed for a short period of time and then moved on to other countries or returned to Ukraine as the situation changed [59]. A short-term stay may not be sufficient for latent TB to develop and be diagnosed in Romania. Any cases which did develop would have been easily treatable if the person moved to another country. This mobility distributes the cases across countries instead of having them focused in the country of arrivals. Finally, Romania’s strong national TB program has experience of case finding and contact investigations. As Ukraine’s TB is well-known, any Ukrainian refugee who presented with symptoms would be assessed for TB; aware clinicians could quickly diagnose and segregate cases, stopping the spread. The combined effect of these determinants was that the expected wave of TB among the Ukrainian refugees did not occur in Romania to a large degree. Countries in which refugees had been more numerous or intensive screening had been used did find more cases (including drug-resistant TB), pointing to the need for continued vigilance and targeted TB services for refugees [36]. However, this was not the case in the Romanian context where the influx of Ukrainian refugees did not correspond to an increase in TB. This fact shows us that with good public health, mass displacement can be achieved without a dramatic increase in communicable diseases. Romania’s experience-probably one of the limited examples worldwide in this context-few TB cases in spite of hundreds of thousands of refugees, clearly shows the protective effects of vaccination, demography and of effective health policy. The focus on high-risk groups and access as opposed to scare-mongering widespread screening is emphasized. In the future, the importance of monitoring and assistance for TB programs (both in host countries and Ukraine itself) will continue to be of concern, particularly to control any cases of latent TB infections or MDR-TB cases that may come into existence. However, the period 2022–2025 represents a cautious optimism for avoiding a public health disaster of TB among Ukrainian refugees in Romania, both due to the favorable circumstances and because of effective interventions.

Early detection, isolation, and treatment of influenza cases are crucial to prevent outbreaks. Administering antiviral medications such as oseltamivir to high-risk patients and ensuring access to healthcare for influenza complications are essential to reduce disease spread [60]. Influenza complications include pneumonia, the worsening of chronic diseases, such as asthma and diabetes, and secondary bacterial infections [61]. Poor nutrition and exposure further increase susceptibility to influenza and pneumonia, particularly in vulnerable groups such as children and older individuals [62]. A study conducted at Suceava Hospital in Romania analyzed the medical conditions of Ukrainian child refugees. Interstitial pneumonia was diagnosed in 23% of pediatric patients, highlighting the vulnerability of refugee children to respiratory infections [42]. In the Department of Infectious Diseases, 29.2% of the total number of hospitalized refugee patients had influenza. All patients responded favorably to oseltamivir and were discharged. Our data are consistent with reports from other European host countries, which describe influenza and COVID-19 as the leading causes of infectious disease consultations among refugees [52,53]. However, unlike Poland, Germany, or the Czech Republic, which documented increases in tuberculosis among Ukrainian nationals [52,53,54], no cases of TB were identified among refugees hospitalized in Suceava during the study period. This aligns with Romania’s national surveillance data, which have not shown a measurable TB increase attributable to refugee influx.

Ukraine has one of the highest HIV prevalence rates in Eastern Europe, with approximately 1% of the population affected as of 2019 [63,64]. Displacement disrupts access to antiretroviral therapy, increasing the risk of transmission and adverse health outcomes [65]. In Romania, refugees living with HIV can register and receive treatment at one of the country’s Regional HIV centers, which offer antiretroviral therapy consistent with Ukrainian protocols, along with the necessary monitoring through specific blood tests. Of the three HIV-positive patients who presented to the Department of Infectious Diseases, two were diagnosed in Ukraine and required clinical–biological re-evaluation and continuation of the antiviral treatment regimen. The third patient was diagnosed with an HIV infection at our department and presented to the emergency department with fever, lymphadenopathy, and the appearance of an eruptive syndrome.

Ukraine experienced significant measles outbreaks before the conflict, partly owing to low vaccination coverage. In 2016, only 31% of the population had received the measles vaccine, leading to outbreaks of over 57,000 cases reported in 2019 [66,67]. None of the patients diagnosed with measles at our department were vaccinated against measles and this emphasizes the need for targeted vaccination campaigns among refugee children. Some of the refugees with measles who were seen in the emergency department presented with interstitial pneumonia, laryngitis, liver cytolysis, or acute dehydration syndrome, with 12.5% requiring hospitalization. In late 2021, cases of vaccine-derived poliovirus type 2 (cVDPV2) were reported in Ukraine, raising concerns about potential outbreaks, especially in areas with low immunization rates [68] and possible transmission in refugee populations [69]. To date, no cases of poliovirus infection have been reported among refugees from Suceava County.

Disruptions in COVID-19 vaccination efforts and crowded living conditions increase the risks of SARS-CoV-2. COVID-19 has had a severe influence on refugees owing to overcrowded living conditions, limited healthcare access, and weak health infrastructure in camps and host communities [70]. The pandemic has worsened the existing health inequalities and created new challenges for displaced populations. Because refugee camps and shelters make social distancing nearly impossible, refugees are at higher risk for COVID-19. Furthermore, limited water supply and sanitation increase the risk of viral spread, and many refugees lack access to testing, treatment, and vaccines [71]. A weakened immune system also makes refugees more vulnerable to severe COVID-19. A lack of proper health education leads to fear, stigma, and vaccine hesitancy [72].

Poor sanitation and lack of clean water contribute to hepatitis A and E outbreaks [73,74,75]. Although cases of acute hepatitis A and viral hepatitis E were recorded in Suceava County between February 2022 and February 2025, none were Ukrainian refugees. Although not endemic in Ukraine, the disruption of sanitation systems increases the risk of cholera and dysentery [76,77]. Norovirus and rotavirus can cause severe diarrhea, particularly in refugee camps [78,79]. Cases of rotavirus enterocolitis have also been recorded in Ukrainian refugee children in Suceava County, 0.6% of them requiring hospitalization. The prognosis was favorable after treatment with rehydration solutions and probiotics, and the digestive symptoms quickly resolved. Overcrowded and unsanitary conditions increase the risks of louse-borne typhus and trench fever. Ukraine has endemic tick-borne diseases, and Suceava County has many forests. Therefore, refugees are at risk of tick bites and developing tick-borne encephalitis and Lyme disease, which could affect displaced populations in forested areas [80,81]. For patients who presented with tick bites, Lyme disease prophylaxis was performed by administering doxycycline, and tick extraction and wound dressing were performed at the emergency department.

Many refugees have chronic conditions requiring treatment, including HIV infection, TB, and hepatitis C [82]. Reduced access to antiretroviral therapy for patients with HIV could lead to increased transmission and worsened health outcomes [83,84]. Pregnant women may face a higher risk of complications owing to a lack of prenatal care [85,86].

The age- and sex-stratified prevalence data (Table 3, Table 4 and Table 5) demonstrate that disease burden among refugees is not homogeneous. Children were disproportionately affected by viral respiratory infections, while adults were more often hospitalized with COVID-19, HIV, or hepatitis C. These findings support the need for differentiated health strategies. In children, we must strengthen vaccination campaigns and ensuring rapid access to rehydration therapy for enteric diseases. For adults, we must maintain access to antivirals for COVID-19, antiretroviral therapy for HIV, and hepatitis C treatment programs. For all refugees, it is important to ensure routine screening and management of comorbidities, which increase vulnerability to infectious disease complications [25]. Recognizing the increased risk of vaccine-preventable diseases, Romania has initiated vaccination drives targeting refugee and local populations. These campaigns focus on measles, polio, influenza and COVID-19, aiming to curb potential outbreaks [87,88,89]. Given Ukraine’s high incidence of TB, Romanian health authorities have implemented screening programs to identify and treat TB among refugees. This proactive approach aids early detection and reduces transmission risks [90]. Romania employs cultural mediators to bridge language barriers and assist refugees in navigating the healthcare system [32]. These mediators facilitate communication between healthcare providers and refugees, ensuring that individuals understand how to access services and the available treatments [91]. Blue dot centers: In collaboration with UNICEF and other organizations, Romania has established “Blue Dot” centers at border crossings and along major migration routes. These centers offer integrated services, including family reunification support, psychological first aid, and information on health and hygiene practices [21,92,93]. With support from international organizations, Romanian authorities have investigated the evolving needs of refugees. These assessments inform policy decisions and help tailor healthcare services to address specific challenges faced by the displaced population [94,95,96,97,98]. Emergency medical services: Hospitals such as the “St. Ioan cel Nou” Clinical Emergency Hospital in Suceava have analyzed the medical conditions prevalent among Ukrainian child refugees. Respiratory diseases, musculoskeletal injuries, and digestive disorders are common, guiding the allocation of medical resources and specialized care [42]. Through these comprehensive measures, Romania aims to safeguard the health of both refugee and host communities, emphasizing the importance of accessible healthcare, effective disease prevention, and continuous support for vulnerable populations.

This study is limited by its single-center design and reliance on hospitalized patient data, which may not fully capture infectious disease prevalence among Ukrainian refugees in Romania. No official data were published on the Romanian Ministry of Health website or in other studies regarding the prevalence of infectious diseases among Ukrainian refugees in Romania. Consequently, the data presented in this study could not be compared with the broader national context of infectious disease cases among refugee patients in Romania or with data from other medical units. Additionally, information on the number of Ukrainian refugee patients diagnosed with infectious diseases by family physicians or primary health centers has not been previously made available. Nonetheless, the use of age- and sex-stratified prevalence tables (Table 3, Table 4 and Table 5) strengthens the epidemiological analysis and provides actionable insights for clinicians and policymakers.

Future needs and recommendations include the long-term integration of Ukrainian refugees into host countries’ healthcare systems, with a focus on strengthening disease prevention and vaccination programs. Refugees should be included in national influenza vaccination programs to prevent outbreaks, and influenza response efforts should be integrated into broader infectious disease programs, such as those targeting TB and COVID-19. Improved medical supply chains are essential for effective chronic disease management, alongside enhanced healthcare system capacities to enable early diagnosis and treatment. Additionally, expanding the use of telemedicine and digital health solutions can help bridge gaps in healthcare access and continuity of care.

## 5. Conclusions

Although displacement resulting from the conflict in Ukraine has led to an increased incidence of infectious diseases, particularly TB, in some host countries, no increase in TB cases has been reported among Ukrainian refugees in Suceava County. The most common infectious pathologies encountered among refugees in the context of the conflict in Ukraine were respiratory diseases such as COVID-19, influenza, respiratory viruses, and measles. Continuous monitoring, robust healthcare responses, and targeted vaccination campaigns are crucial for mitigating the spread of these diseases among refugee populations and host communities. The response in Suceava County to the Ukrainian refugee crisis exemplifies a multifaceted approach that combines immediate relief and long-term integration. Through the collaborative efforts of local communities, organizations, and international partners, the region continues to deliver essential support to those affected by the conflict.

## Figures and Tables

**Table 1:** Programs and Initiatives for Pregnant Women.

Program/Organization	Services Offered for Pregnant/New Mothers	Examples/Notes
Independent Midwives Association (AMI)—“Refugee Health Journey”	Midwifery services; reproductive health (including contraception, prenatal and postnatal care); monitoring of pregnancy and newborn; psychological counselling; support for lactation; helping refugees (pregnant, new mothers, single mothers) navigate the health system; translation/interpreting; family planning [22].	From September 2023–February 2024, AMI provided these services to over 4000 people (women & vulnerable persons) under this project funded by CORE and American Red Cross.
UNICEF + Independent Midwives Association	Counselling in early months after childbirth; training for birth, breastfeeding, baby care; outreach for mothers; help navigating health services; vaccination for children; legal/translation support [23].	By March 2023, nearly 1400 refugee mothers + their children had benefited through the joint project “Support network for reproductive health and increased access to pediatric care for refugee women and children.”
Blue Dot Centers (UNICEF/UNHCR, etc.)	Safe spaces dedicated to mothers & babies; counselling; referrals for health issues; first aid/nutrition/hygiene; baby food and sanitary kits; information; psychological support [23].	Blue Dot hubs are located at border crossing points and cities (e.g., Sighetu Marmației, Siret, Isaccea, Albița, Huși, Iași, Brașov, Bucharest) to ensure access soon after arrival.
MSD and Save the Children Romania	Project to offer emergency care for pregnant Ukrainian refugee women and newborns [24].	The partnership provides access to emergency obstetric care and newborn support. Details include ensuring that pregnant women have safe access to delivery services, likely covering medical costs, transport, etc.
Housing/Accommodation for Pregnant Women	Some initiatives provide dedicated housing or prioritize pregnant women in shelters or temporary accommodation; arranging safer, more accessible housing.	For example, the Independent Midwives Association “has partnered with a group of ~50 mothers to secure housing for pregnant women, women with small children and children with special needs”, including access to antenatal care, legal and translation support. https://internationalmidwives.org/midwives-associations-leading-efforts-across-europe-to-address-unmet-needs-of-women-and-their-families (Accessed on 25 August 2025).
Government Health System Support	Under national policies, refugees have access to healthcare (including maternity), maternal monitoring, prenatal check-ups, childbirth under public health systems similar to citizens (though some obstacles exist). Also, the integration of midwife-based services via NGOs to cover gaps has taken place [25].	

**Table 2:** Ukrainian Refugees in Key Host Countries (2024–2025).

Host Country	Refugees Hosted (Approx.)	Healthcare Access	Economic/Employment Situation	Legal Status and Social Protection
Germany	~1.2 million under Temporary Protection (July 2025)	Full access to public health insurance once registered: includes chronic & maternity care. Interpretation and waiting times are issues.	Many employed (~50% of working-age in surveys), but often in jobs below qualification; wage gaps compared with locals.	EU Temporary Protection Directive (TPD): residence, work, housing, education, benefits.
Poland	~1.0 million (July 2025)	Entitled to public healthcare equal to nationals; language and overload of system reported as barriers.	High employment, often women in services/retail; income below host average. Some reliance on aid.	TPD rights; Poland also offers national ID (PESEL UKR) for access to health, social aid, schooling.
Czechia	~0.37 million (July 2025)	Emergency and basic healthcare free; longer-term care requires insurance enrolment. Capacity constraints exist.	Higher share of refugees working than many EU states; many in manual/low-paid roles.	TPD protections; simplified access to jobs, schools, and certain welfare benefits.
Romania	~90,000–100,000 refugees (2024–2025)	Free access to national health services (including maternity and pediatric); NGOs fill gaps (midwives, translation, psychosocial).	Lower employment integration; many rely on NGO/government housing/food schemes.	TPD rights; national 50/20 program (later cash allowances) for housing and food.
Moldova (non-EU)	~100,000–110,000 (2024)	Limited resources; basic emergency care available, with UN/NGO help for chronic, maternal and child health.	Very limited job market; refugees highly dependent on aid.	Not under EU TPD, but Moldova grants temporary protection and humanitarian assistance with UNHCR support.

**Table 3:** Infectious diseases diagnosed among Ukrainian refugees in “St. Ioan Cel Nou,” Suceava (n = 672).

Condition	n	Prevalence (%)
COVID-19	208	31.0
Upper respiratory tract infection	192	28.6
Influenza	132	19.7
Acute enterocolitis	77	11.5
Measles	32	4.8
Mononucleosis	6	0.9
HIV complications	3	0.5
Tick bites	8	1.2
Dog bites (rabies prophylaxis)	12	1.8

Note. Values are expressed as absolute numbers and prevalence (% of consultations).

**Table 4:** Demographic characteristics of hospitalized Ukrainian refugees (n = 48).

Age Group (Years)	n	% of Total	Male, n (%)	Female, n (%)
0–4	13	27.1	10 (20.8)	3 (6.2)
5–14	11	22.9	8 (16.7)	3 (6.2)
15–18	2	4.2	1 (2.1)	1 (2.1)
Children (0–18)	26	54.2	19 (39.6)	7 (14.6)
19–34	6	12.5	3 (6.2)	3 (6.2)
35–44	4	8.3	2 (4.2)	2 (4.2)
45–54	4	8.3	2 (4.2)	2 (4.2)
55–64	2	4.2	1 (2.1)	1 (2.1)
65–74	4	8.3	2 (4.2)	2 (4.2)
>75	2	4.2	1 (2.1)	1 (2.1)
Adults (≥19)	22	45.8	10 (20.8)	12 (25.0)
Total	48	100	29 (60.4)	19 (39.6)

Note. Percentages may not total 100 due to rounding.

**Table 5:** Prevalence of infectious diseases among hospitalized Ukrainian refugees (n = 48), stratified by age and sex.

Disease	Children (n = 26)	Adults (n = 22)	Males (n = 20)	Females (n = 28)	Total Prevalence (%)
COVID-19	6 (23.1)	9 (40.9)	8 (40.0)	7 (25.0)	15 (31.2)
Influenza	12 (46.2)	2 (9.1)	9 (45.0)	5 (17.9)	14 (29.2)
Measles	2 (7.7)	2 (9.1)	2 (10.0)	2 (7.1)	4 (8.3)
Varicella	1 (3.8)	0 (0.0)	1 (5.0)	0 (0.0)	1 (2.1)
Rotavirus enterocolitis	4 (15.4)	0 (0.0)	3 (15.0)	1 (3.6)	4 (8.3)
C. difficile enterocolitis	0 (0.0)	4 (18.2)	3 (15.0)	1 (3.6)	4 (8.3)
HIV infection	0 (0.0)	3 (13.6)	3 (15.0)	0 (0.0)	3 (6.2)
Chronic hepatitis C	0 (0.0)	2 (9.1)	0 (0.0)	2 (7.1)	2 (4.2)
Sepsis	0 (0.0)	1 (4.5)	1 (5.0)	0 (0.0)	1 (2.1)

Note. Values are expressed as n (%) within each subgroup. Total prevalence (%) is based on the full cohort (n = 48).

**Table 6:** Average length of stay among hospitalized Ukrainian refugees by diagnosis (n = 48).

Diagnosis	n	Mean Length of Stay (Days)	SD (Days)
COVID-19	15	6.3	2.1
Influenza	14	4.1	1.0
Measles	4	7.5	3.8
Varicella	1	5.0	–
Rotavirus enterocolitis	4	3.5	0.6
C. difficile enterocolitis	4	6.8	5.0
HIV infection	3	5.0	2.6
Chronic hepatitis C	2	2.5	0.7
Sepsis	1	13.0	–

Note. Values represent mean ± standard deviation (SD) where applicable; SD not calculated for single cases.

## Data Availability

The original contributions presented in this study are included in the article. Further inquiries can be directed to the corresponding author(s).

## Abbreviations

The following abbreviations are used in this manuscript:

AMI	Independent Midwives Association
BCG	Bacillus Calmette-Guerin
*Cl. difficile*	*Clostridioides difficile*
cVDPV2	vaccine-derived poliovirus type 2
HIV	human immunodeficiency virus
WHO	World Health Organization
IOM	International Organization for Migration
MDR-TB	multi-drug-resistant TB
MHPSS	mental health and psychosocial support
NCD	noncommunicable diseases
NGO	non-governmental organization
RIF	Innovation and Resilience Center
TB	tuberculosis
Tdh	Terre des Hommes
TPD	Temporary Protection Directive
UNHCR	Office of the United Nations High Commissioner for Refugees
UNICEF	United Nations Children’s Fund
